# Three-dimensional assessment of pharyngeal airway hyoid bone and craniocervical changes after stabilization splint treatment in temporomandibular disorder patients

**DOI:** 10.1038/s41598-025-17583-0

**Published:** 2025-09-25

**Authors:** Pengyu Chen, Saba Ahmed Al-hadad, Chenyu Rao, Yi Li, Chunshen Li, Enas Senan ALyafrusee, Barakat Al-Tayar, Ibtehal Almagrami, Leena Ali Al-Warafi, Xi Chen, Yunshan Zhao

**Affiliations:** 1 Department of Stomatology, The First Affiliated Hospital of Xi’an Jiaotong University, Xian, 710061 Shaanxi People’s Republic of China; 2 Department of Orthodontics and Dentofacial Orthopedics, Faculty of Dentistry, Ibb University, Ibb, Republic of Yemen; 3 West China Hospital of Stomatology, Sichuan University, Chengdu, Sichuan People’s Republic of China; 4 Department of Orthodontics, School of Stomatology, China Medical University, Shenyang, People’s Republic of China; 5 Orthodontics Division, Faculty of Medicine and Health Sciences, Taiz University, Taiz, Yemen; 6 Department of Orthodontics, Faculty of Dentistry, First Affiliated Hospital of Zhengzhou University, Henan, China

**Keywords:** Cone-Beam computed tomography, Hyoid bone, Occlusal splints, Parapharyngeal space, Temporomandibular joint disorders, Three-Dimensional, Health care, Medical research

## Abstract

Stabilization splint (SS) therapy is commonly used to treat temporomandibular disorders (TMD), but its three-dimensional (3D) effects on pharyngeal airway (PA) dimensions, hyoid bone (HB) position, and craniocervical (CC) posture remain unclear. This study primarily aimed to evaluate the 3D changes in the PA, with a secondary focus on the HB and CC posture, in adult TMD patients after SS therapy. This retrospective study analyzed 80 adult TMD patients (mean age: 23.88 ± 5.8 years) treated with SS for 6–12 months. Pre- and post-treatment cone-beam computed tomography (CBCT) scans were used to measure 3D changes in PA (naso-, oro-, hypo-, and total pharyngeal spaces), HB position, and CC posture using InVivo and Dolphin software. Statistical analysis included paired t-tests and Wilcoxon signed-rank test (*P* < 0.05). Significant reductions occurred in oropharyngeal surface area and sagittal width (*P* = 0.021; *P* = 0.017, respectively) and hypopharyngeal parameters (surface area, volume, minimal constriction area, sagittal width; *P* < 0.05). HB measurements showed significant decrease in hyoid-mandibular plane (H-MP), hyoid-retrognathia distances (H-RGN) and hyoid triangle height measurements (*P* = 0.002, *P* = 0.014 and *P* = 0.029, respectively), with a significant increase in retrognathia-sella distance (RGN-S, *P* = 0.003). CC posture showed significant decreases in the Sella–Nasion to odontoid process tangent angle (SNL-OPT) and to the cervical vertebra tangent angle (SNL-CVT) (*P* = 0.005, *P* = 0.003, respectively). SS therapy significantly reduced oropharyngeal and hypopharyngeal airway dimensions, changed HB position, and improved CC posture post-treatment. These results highlight the multidimensional impact of SS therapy and support incorporating airway and CC posture assessments into TMD management which enhances diagnosis and therapeutic outcomes.

**Supplementary Information:**

The online version contains supplementary material available at 10.1038/s41598-025-17583-0.

## Introduction

The pharyngeal airway (PA) is a tubular structure that extends from the skull base to the sixth cervical vertebra, bounded posteriorly by the spine and anteriorly by the nasal septum, mandible, and hyoid bone (HB)^1^. A retrusive mandible or craniofacial deformities can narrow the PA, increasing the risk of obstructive sleep apnea (OSA). Mandibular positioning significantly influences PA morphology by altering the position of the HB, which, along with the tongue base and soft palate, plays a crucial role in airway stability^1^. Several studies have demonstrated that mandibular advancement appliances, such as Forsus Fatigue Resistance Device (FFRD) and Twin Block (TWB)^2^can induce significant skeletal and pharyngeal changes in Class II patients, including anterior displacement of the HB and expansion of the oropharyngeal airway^2,3^. Additionally, in Class III malocclusion, Frankel III appliance therapy has been shown to increase upper airway dimensions despite posterior mandibular repositioning^4^.

The position of the HB is dynamic, influenced by cervical spine curvature and the contraction of infra- and suprahyoid muscles, allowing it to adapt to changes in neck and head posture^5^. The angulation of the craniocervical (CC) region also plays a critical role, as a hyperextended head posture can alter PA dimensions^6^. Anterior mandibular shifts can affect not only the HB but also tongue position, cervical vertebrae alignment, and overall PA space^7^. Clinical studies have further linked temporomandibular disorders (TMD) to cervical spine dysfunction, with patients frequently reporting concurrent neck pain. Temporomandibular joint (TMJ) disc displacement has been shown to alter HB position and CC posture, suggesting a bidirectional influence between craniomandibular and CC systems^8^. Treatment approaches for TMD vary, ranging from invasive interventions (e.g., TMJ surgery) to minimally invasive therapies (e.g., corticosteroid injections) and noninvasive methods such as occlusal splints. Stabilization splints (SS) are among the most commonly used noninvasive treatments for TMD^9^with several studies demonstrating their effectiveness in reducing pain, mitigating sleep bruxism, managing muscular TMD, and promoting condylar remodeling^10–13^.

Numerous studies have indicated that the use of dental appliances leads to changes in the PA, HB, and CC due to alterations in mandibular positioning or other factors. However, most of the existing research has focused on functional or orthopedic appliances, with comparatively limited attention given to SS, despite their widespread use in TMD management. Given the anatomical and functional connections among the PA, HB, and CC posture, evaluating the impact of SS therapy on these structures is clinically important. Such an investigation may enhance our understanding of the broader physiological effects of SS and support more comprehensive treatment planning for TMD. To our knowledge, a detailed, three-dimensional (3D) evaluation of changes in PA dimensions, HB position, and CC posture with a larger sample size following SS treatment has not been comprehensively investigated in adult TMD patients. Therefore, the primary aim of this study was to evaluate the 3D changes in the PA, with a secondary focus on the HB and CC posture, following SS treatment in adult individuals with TMD.

## Materials and methods

### Study design

This retrospective clinical study was conducted at the First Affiliated Hospital of Xi’an Jiaotong University, located in Xi’an, Shaanxi Province, China, in accordance with the ethical principles of the Declaration of Helsinki. Prior to data collection and analysis, the study protocol received ethical approval from the Institutional Review Board (Approval No. XJTU1AF2022LSK-027). All participants provided written informed consent prior to participation.

### Sample size calculation

Data were collected from the complete medical records of patients diagnosed and treated between July 2017 and July 2024. Cone beam computed tomography (CBCT) scans, originally obtained for TMJ diagnosis, were also used to assess changes in PA, HB, and CC after SS treatment. The required sample size was calculated using G*Power software (Version 3.1.3, University of Kiel, Germany) with an alpha of 0.05 and 80% power. This calculation based on the findings from Derwich et al.^5^ study, who reported a mean change of 7.8 ± 6.4 mm in the hyoid triangle measurement (H-H’ distance), and 8.3 ± 2.6 mm in the oropharyngeal lower width after occlusal splint therapy. Power analysis indicated that a minimum of 55 subjects was required based on H-H’ distance changes, and 62 subjects based on oropharyngeal lower width changes; the final sample size was increased to at least 80.

### Selection criteria

The SS intervention process followed our previously published protocol^14,15^. Inclusion criteria were: adults (≥ 18 years) with complete medical and dental records; TMD diagnosis per DC/TMD criteria, including muscle and/or TMJ pain, mandibular range of motion, TMJ noises, and confirmed disc displacement with reduction (based on clinical evaluation) and/or myalgia; compliance with treatment; use of maxillary SS without skeletal mandibular asymmetry;; SS retention eligibility to ensure proper splint retention throughout the treatment (defined as fully erupted permanent dentition excluding third molars, stable occlusal contacts without severe malocclusion [e.g., crossbites, extreme crowding], and healthy periodontal status with no active disease and adequate bone support); normal Body Mass Index (18.5–24.9 kg/m²)^16^; and completed SS treatment with pre- and post-treatment CBCT scans. Exclusion criteria included: history of craniomaxillofacial trauma; active idiopathic condylar resorption; chronic upper respiratory issues (e.g., nasopharyngeal disorders; adenoid or tonsillar hypertrophy); head or neck trauma; prior orthognathic/orthodontic/TMJ surgery; and metabolic or systemic immune disorders.

### CBCT assessment

**Fig. 1:**
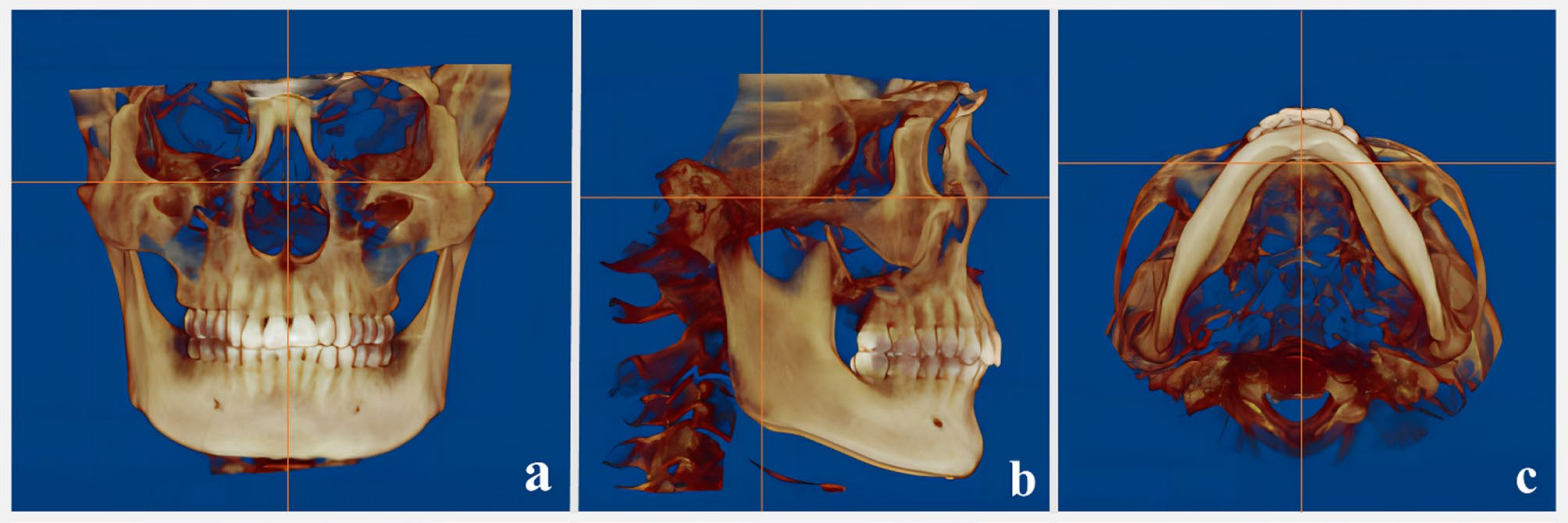
3D reconstruction of the head position. (images generated using Dolphin 11.95; https://www.dolphinimaging.com).

3D images were acquired using a CBCT device (KaVo 3D eXam; KaVo Dental, Bismarckring, Germany) with settings of 120 kV, 37.1 mA, a 23 × 17 cm field of view, 0.3 mm voxel size, 17.8 s exposure time, and 0.3 mm slice thickness. Participants were instructed to sit upright with teeth in maximum intercuspation, the Frankfort horizontal plane parallel to the floor, and the midsagittal plane perpendicular to it; they were instructed to avoid swallowing during the scan. CBCT scans were obtained at two-time points: before the initiation of SS therapy (baseline) and after the completion of the treatment (post-treatment), with treatment duration ranging from 6 to 12 months. Both pre- and post-treatment CBCT scans were acquired without the SS in place to ensure evaluation of the natural anatomical position. CBCT data were converted to Digital Imaging and Communications in Medicine file format and analyzed using InVivo 6.0.3 (Anatomage, San Jose, CA) for HB and CC measurements, and Dolphin 11.95 (Dolphin Imaging, Chatsworth, CA) for PA measurements and segmentation. Prior to measurements, the head positions were reconstructed and standardized for all patients (Fig. 1).

### Three-dimensional assessment

**Fig. 2:**
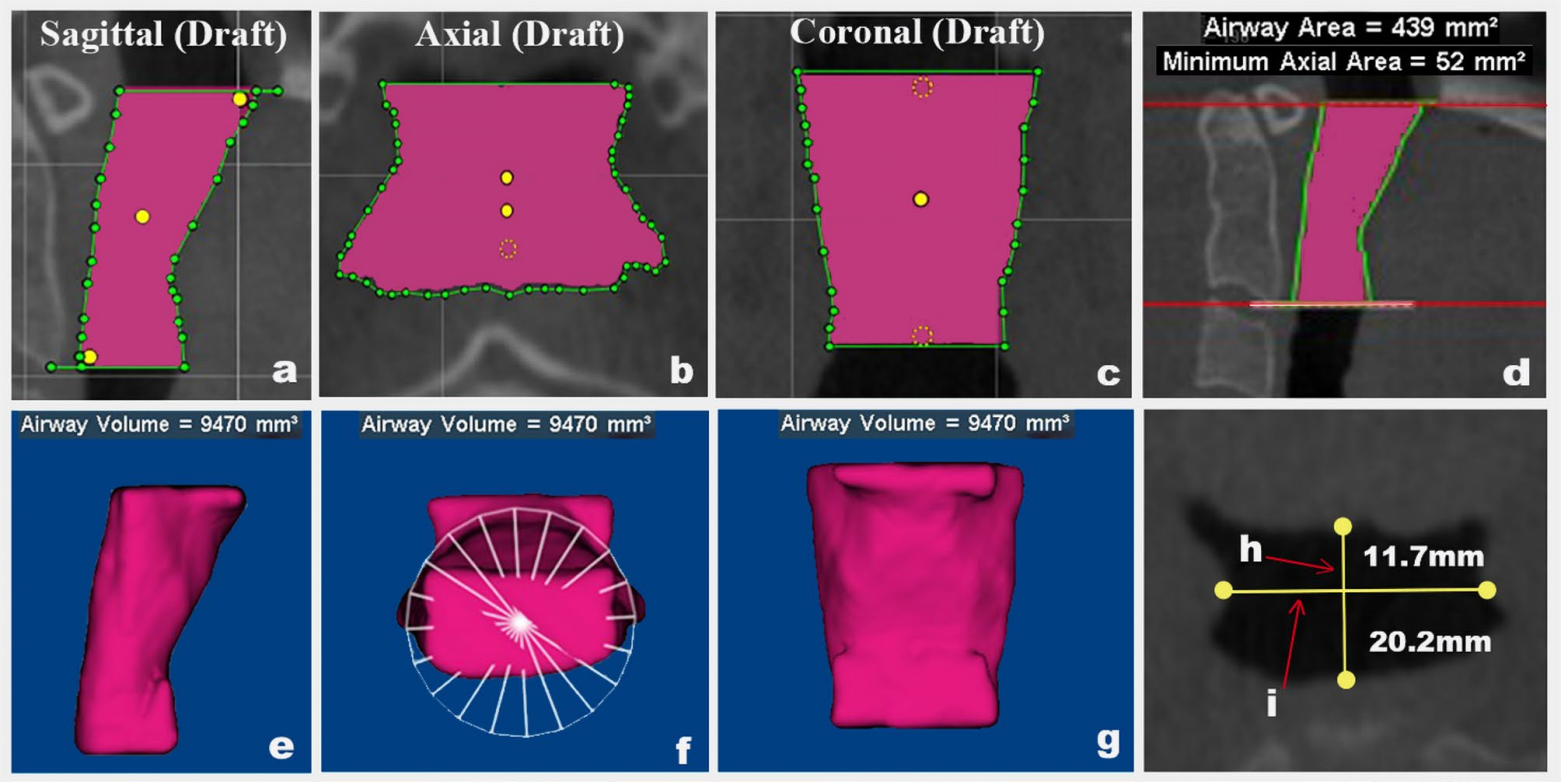
Oropharyngeal airway: (**a**) Sagittal view of the surface area; (**b**) Axial view of the surface area; (**c**) Coronal view of the surface area; (**d**) Multiplanar view of the airway area and MCA; (**e**) Sagittal view of the airway volume; (**f**) Axial view of the airway volume; (**g**) Coronal view of the airway volume. (**h**) Oropharyngeal sagittal width OP (A/P); (**i**) Oropharyngeal lateral width OP (R/L). (images generated using Dolphin 11.95; https://www.dolphinimaging.com).

**Fig. 3:**
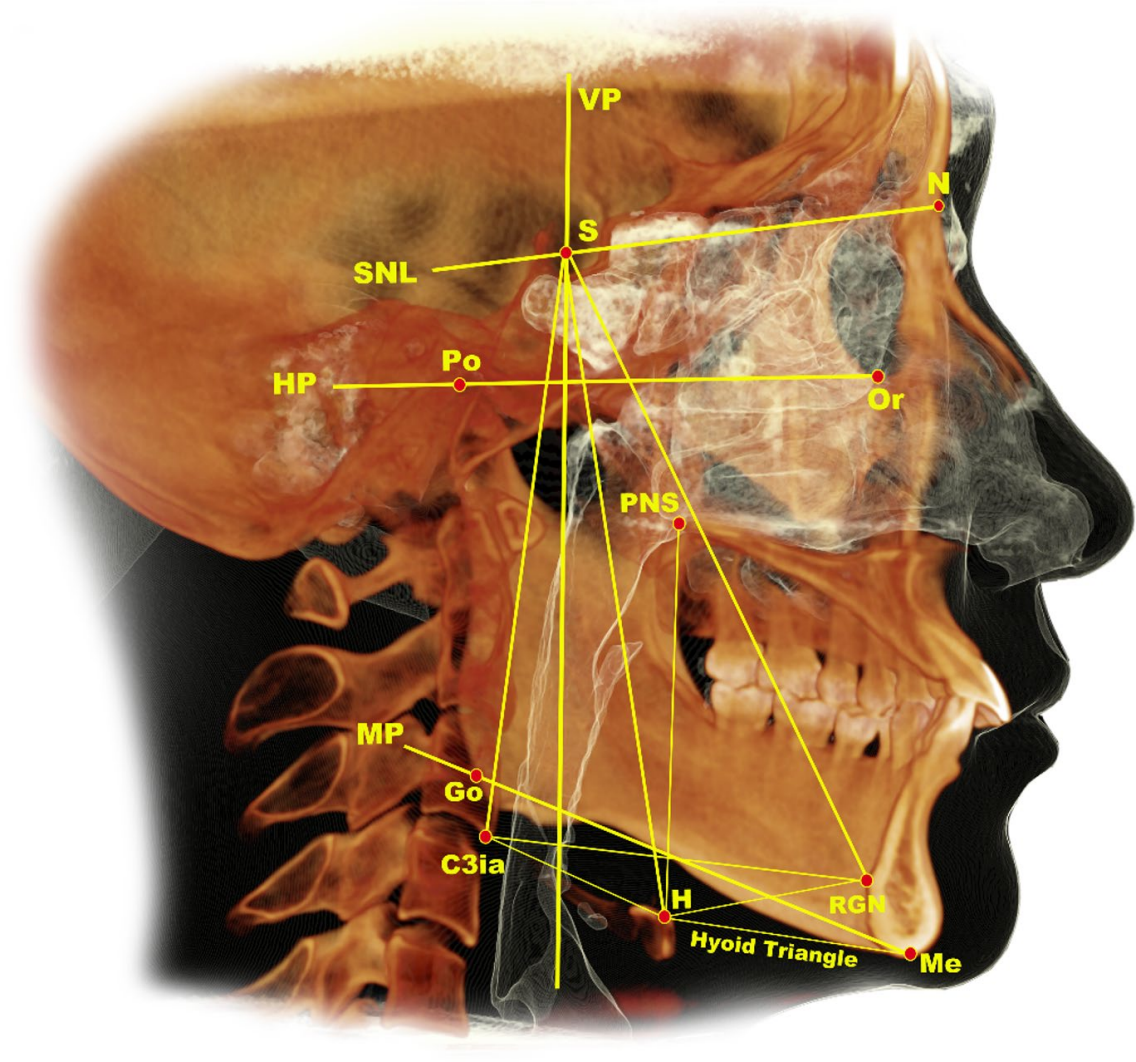
Anatomical reference points and measurements of the hyoid bone. (image generated using InVivo 6.0.3; https://dexis.com/en-us/software-invivo-6).

**Fig. 4:**
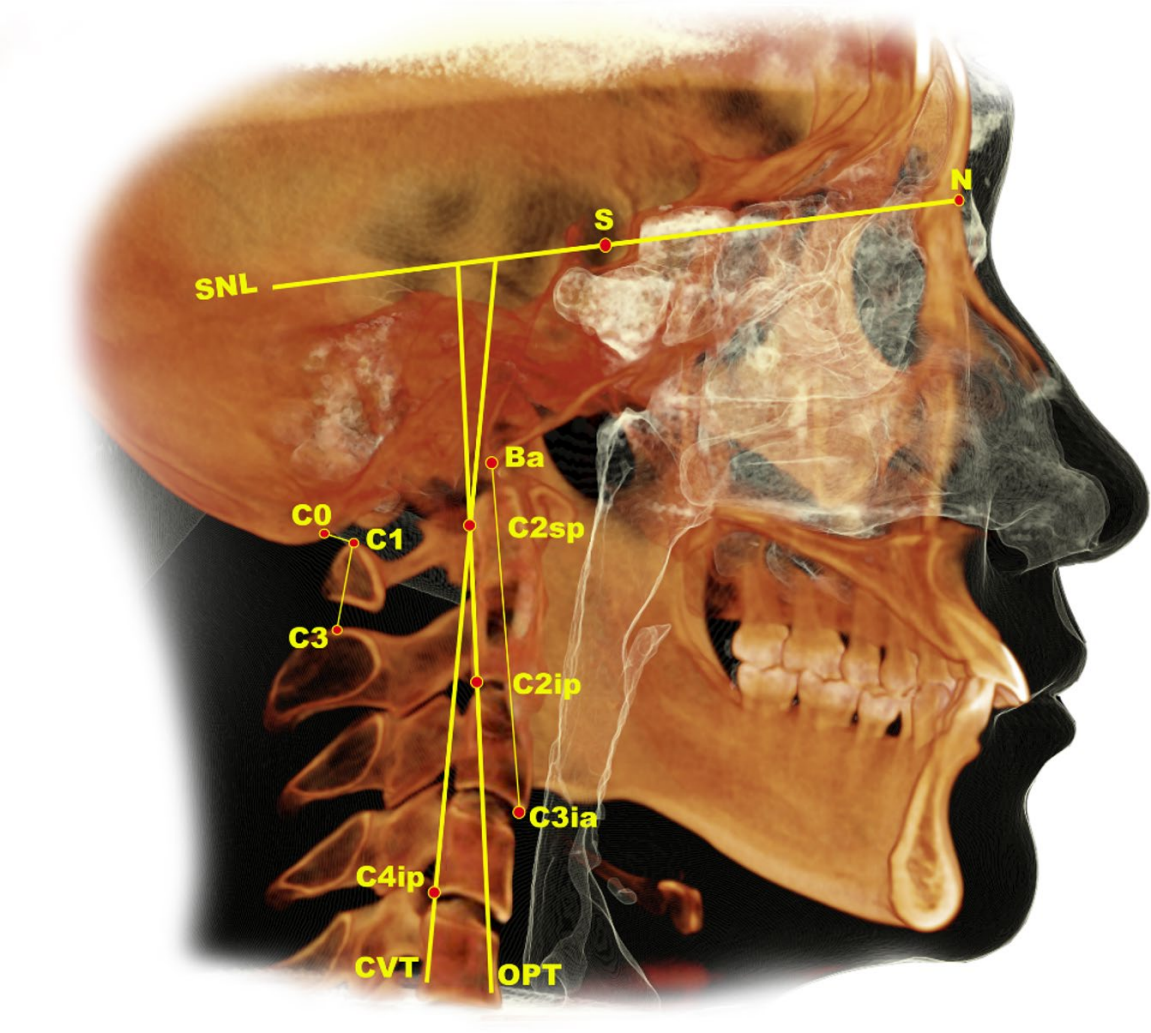
Anatomical reference points and measurements of the craniocervical region (image generated using InVivo 6.0.3; https://dexis.com/en-us/software-invivo-6).

Table 1 outlines the PA, CC, and HB landmarks, reference planes, lines, and PA boundaries, while Table 2shows PA dimension measurements, in which the measurement method follows Al-Somairi et al.,^1,17^. Figure 2 shows oropharyngeal airway measurements, while Supplementary Figs. 1–3 show measurements for the nasopharyngeal, hypopharyngeal, and total PA. The airway module was used for segmentation, selecting slices aligned with the midsagittal plane for optimal airway visualization. Seed points expanded the PA, with sensitivity set to 72–73 per previous studies^2,17^. Chipping boundaries constrained expansion, combining manual and automated segmentation for optimal accuracy^17^. Table 2also includes HB measurements according to Mohamed et al.,^18^. (Fig. 3) and CC measurements as described by Kang et al.^19^ (Fig. 4).

**Table 1:** Definitions of the reference landmarks, lines, planes and pharyngeal airway borders.

	Name	Abbreviation	Definition
Anatomical Landmarks	Nasion	N	The most anterior and midpoint of the nasofrontal structure
	Sella	S	Central point of the sella turcica
	Basion	Ba	The lowest and most posterior portion of the foramen magnum, located along the skull’s midline
	Left/Right Orbitale	Or	The lowest and middle point on the lower edge of the orbit on the right or left side
	Left/Right Porion	Po	The highest point of the external auditory meatus on the right or left side
	Menton	Me	The lowest midpoint on the bony outline of the mandibular symphysis, at the chin
	Left/Right Gonion	Go	The midpoint of the angle formed by the intersection of the ramus line and the mandibular body line
	Posterior nasal spine	PNS	The middle of the palatine bone’s distal end
	Retrognathion	RGN	Refers to the most prominent point located at the back of the mandibular symphysis
	Hyoid bone	H	The highest and most forward point of the hyoid bone
	C0 point	C0	Base of the occipital
	C1 point	C1	The posterior arch of Atlas
	C2 point	C2	Spinous process of the second vertebra
	C2ip point	C2ip	The lowest and backmost point of the second vertebra
	C2sp point	C2sp	The most superior and posterior point of the second vertebra
	C3ia point	C3ia	The lowest and frontmost point of the third vertebra
	C3ip point	C3ip	The lowest and backmost point of the third vertebra
	C4ia point	C4ia	The lowest and frontmost point of the fourth vertebra
	C4ip point	C4ip	The lowest and backmost point of the fourth vertebra
	Roof	R	Sagittally, the peak of the pharyngeal airway that is positioned at the top of the nasopharynx within the MSP represents the highest point
	Nasopharyngeal posterior and anterior points	NP (P/A)	The most anterior (NP- A) and posterior points (NP- P) in the PNS plane are in the axial view
	Oropharyngeal posterior and anterior points	OP (P/A)	The most anterior (OP- A) and posterior points (OP- P) in the C2 plane are in the axial view
	Hypopharyngeal posterior and anterior points	HPP (P/A)	The most anterior (HPP- A) and posterior points (HPP- P) in the C3 plane are in the axial view
	Nasopharyngeal left and right lateral points	NP (L/R)	The most lateral left (NP- L) and lateral right (NP- R) points in the PNS plane in the axial view
	Oropharyngeal left and right lateral points	OP (L/R)	The most lateral left (OP- L) and lateral right (OP- R) points in the C2 plane in the axial view
	Hypopharyngeal left and right lateral points	HPP (L/R)	The most lateral left (HPP- L) and lateral right (HPP- R) points in the C3 plane in the axial view
Reference line and planes	Midsagittal plane	MSP	Passing through the points N, Ba, and S
	Frankfurt horizontal plane	FH	Extended through the left and right Po along with the right Or
	Vertical plane	VP	Passing through the sella point, it is oriented perpendicularly to the MSP and FH planes
	Mandibular plane	MP	Marked by three landmarks: the gnathion, and the right and left gonion
	OPT line	OPT	It traverses the inferior C2ip and aligns with the posterior tangent of the odontoid process (C2sp).
	CVT line	CVT	It traverses the inferior C4ip and aligns with posterior tangent of the odontoid process (C2sp).
	Mid-C3ia-RGN line	Mid-C3ia-RGN	Positioned at the center of the hyoid triangle, midway between RGN and C3ia
	Posterior nasal spine plane	PSN Plane	Passing through PNS, it represents and runs in parallel alignment with the FH plane
	2nd cervical vertebra plane	C2 Plane	Passing through C2, it represents and runs parallel to the FH plane
	3rd cervical vertebra plane	C3 Plane	Passing through C3, it represents and runs in parallel alignment with the FH plane
	Sella–Nasion line	SNL	The line extends between points S and N
Pharyngeal airway borders	NP anterior border		Crossing the PNS point perpendicularly to FH plane
	NP inferior border		Aligned parallel to HP, passing through the PNS and perpendicularly to the MSP
	OP superior border		The lower boundary of the NP
	OP inferior border		Intersecting the most front-lower point of C2a, aligned with palatal plane
	HPP superior border		The lower boundary of the OP.
	HP inferior border		Intersecting the most front-lower point of C3a, aligned with palatal plane
	Posterior border		The posterior surface of the pharyngeal airway

**Table 2:** Definitions of parameters related to the pharyngeal airway, hyoid bone and craniocervical.

Measurements		Name	Definition
Nasopharyngeal	Area (mm^2^)	NP-A	Positioned between R point and the plane of the PNS within the MSP
	Volume (mm^3^)	NP-V	Within the MS plane, the volume calculated extends from the PNS plane to the R point
	Minimum constriction area (mm^2^)	NP-MCA	The narrowest region of the nasopharyngeal airway
	Sagittal width (mm)	NP(A-P)	In the axial view, a line is drawn between NP-A and NP-P along the PNS plane
	Lateral width (mm)	NP(L-R)	In the axial view, a line is drawn between the NP-L and NP-R points along PNS plane
Oropharyngeal	Area (mm^2^)	OP-A	Within the MP plane, the measurement extends from the PNS to the C2 plane
	Volume (mm^3^)	OP-V	In sagittal, axial, and coronal views, the measurement extends from the PNS to the C2 plane
	Minimum constriction area (mm^2^)	OP-MCA	The narrowest region of the oropharyngeal airway
	Sagittal width (mm)	OP (A-P)	In the axial perspective, a line is traced between HPP-P and HPP-A on the C3 plane
	Lateral width (mm)	OP (L-R)	In the axial perspective, a line is traced between OP-R and OP-L on the C2 plane
Hypopharyngeal	Area (mm^2^)	HPP-A	Within the MS plane, the measurement extends from the C2 plane to the C3 plane
	Volume (mm^3^)	HPP-V	In sagittal, axial, and coronal perspectives, measurements extend from C2 plane to C3 plane
	Minimum constriction area (mm^2^)	HPP-MCA	The narrowest region of the hypopharyngeal airway
	Sagittal width (mm)	HPP (A-P)	In the axial perspective, a line is traced between HPP-P and HPP-A on the C3 plane
	Lateral width (mm)	HPP (L-R)	In the axial perspective, a line is drawn between HPP-R and HPP-L along the C3 plane
Total pharyngeal	Area (mm^2^)	TP-A	Within the MS plane, the measured area extends from the nasopharyngeal roof to the C3 plane
	Volume (mm^3^)	TP-V	In the midsagittal perspective, the volumetric measurement extends from the nasopharyngeal roof to the C3 plane
	Minimum constriction area (mm^2^)	TP-MCA	The narrowest region of the whole pharyngeal airway
Hyoid bone	Distance of H-RGN (mm)	H-RGN	The distance measured between RGN and H points
	Distance of H-Me (mm)	H-Me	The distance measured between Me and H points
	Distance of H-C3ia (mm)	H-C3ia	The distance measured between C3ia and H points
	Distance of H-PNS (mm)	H-PNS	The distance measured between PNS and H points
	Distance of H-S (mm)	H-S	The distance measured between S and H points
	Distance of H-HP (mm)	H-HP	The distance measured between HP and H points
	Distance of H-VP	H-VP	The distance measured between VP and H points
	Distance of H-MP (mm)	H-MP	The distance measured between MP and H points
	Distance of H-MSP (mm)	H-MSP	The distance measured between MSP and H points
	Distance of RGN-C3ia (mm)	RGN-C3ia	The distance measured between RGN and C3ia points
	Distance of C3ia-S (mm)	C3ia-S	The distance measured between C3ia and S points
	Distance of RGN-S (mm)	RGN-S	The distance measured between RGN and S points
	Height of the hyoid triangle (mm)	H- Me-C3ia	The vertical or linear measurement from the H point to its perpendicular projection on the RGN-C3ia line
Craniocervical	SNL -OPT angle (°)	SNL-OPT	The angle formed between the NSL (Sella -Nasion line) and the OPT line
	SNL -CVT angle (°)	SNL-CVT	The angle formed between the NSL (Sella -Nasion line) and the CVT line
	OPT –CVT angle (°)	OPT–CVT	The angle formed between the CVT and OPT lines
	Basion–C3ia distance (mm)	Ba–C3ia	The distance measured between C3ia and Ba
	Distance of cranium-atlas (mm)	C0–1	The distance measured between C1 and C0
	Distance of atlas-axis (mm)	C1 – C2	The distance measured between C2 and C1

°(degree); mm (millimeters); mm^2^ (millimeters square); mm^3^ (millimeters cubic).

### Statistical analysis

Statistical analysis was performed using SPSS version 26.0 (IBM Corp., Armonk, NY, USA). The normality of the data distribution was evaluated using the Shapiro–Wilk test. The paired t-test and Wilcoxon signed-rank test were applied to compare HB, PA, and CC measurements between pre- and post-treatment. Statistical significance was set at a *P*-value of less than 0.05. The reliability of CBCT measurements, assessed using the intraclass correlation coefficient, was evaluated by two observers who reanalyzed 20 randomly selected cases within a 2-week interval to determine intra- and interexaminer agreement.

## Results

A total of 80 TMD adult patients (average age: 23.88 ± 5.8 years; 28 males and 52 females) were included in this study. According to the DC/TMD diagnostic criteria, the distribution of TMD diagnoses was as follows: myalgia (*n* = 40), arthralgia (*n* = 25), and disc displacement with reduction (*n* = 15). The patients underwent SS therapy for a treatment period that varied between 6 and 12 months, with a mean of 9.8 months. Table 3 shows the pre- and post-treatment measurements of PA spaces, including naso-, oro-, hypo-, and total PA. Table 4 shows HB and CC measurements. Excellent intra- and interobserver reliability was achieved for all the measurement outcomes, with inter- and intraobserver reliability values greater than 0.92.

**Table 3:** Preand posttreatment comparison of pharyngeal airway dimensions.

Measurement		Pre-treatment		Post-treatment		*P*-value
		Mean	SD	Mean	SD	
**Nasopharyngeal**	Area (mm^2^)	220.93	65.57	224.24	62.52	0.314
	Volume (mm^3^)	5838.68	1857.72	5962.89	1863.82	0.275
	MCA (mm^2^)	41.01	23.11	41.96	21.83	0.577
	Sagittal width (mm)	18.63	2.98	18.85	2.66	0.206
	Lateral width (mm)	25.18	3.83	25.50	3.86	0.260
**Oropharyngeal**	Area (mm^2)^	494.93	110.55	480.21	107.83	0.021^*^
	Volume(mm^3^)	12691.53	4375.85	12427.26	4129.96	0.350
	MCA (mm^2^)	96.41	76.52	91.48	73.02	0.232
	Sagittal width (mm)	10.56	2.79	10.10	2.73	0.017^*^
	Lateral width (mm)	24.84	6.34	24.44	6.31	0.264
**Hypopharyngeal**	Area (mm^2^)	185.76	53.87	174.98	56.87	0.003^**^
	Volume (mm^3^)	4957.04	2018.00	4524.50	2318.60	0.006^**^
	MCA (mm^2^)	103.44	57.63	86.85	44.58	0.001^***^
	Sagittal width (mm)	12.74	3.03	11.97	3.30	0.014^*^
	Lateral width (mm)	29.84	4.51	29.69	4.60	0.641
**Total**	Area (mm^2^)	897.31	169.62	882.55	156.84	0.117
	Volume (mm^3^)	24121.86	6204.61	23912.23	6836.14	0.627
	MCA (mm^2^)	36.23	23.88	34.98	22.49	0.219

mm (millimeters); mm^2^ (millimeters square); mm^3^ (millimeters cubic); SD, standard deviation ^*^P < 0.05; ^**^P < 0.01; ^***^P < 0.001.

**Table 4:** Pre-and post-treatment comparison of the hyoid bone position and craniocervical posture.

	Measurement	Pre-treatment		Post-treatment		*P*-value
		Mean	SD	Mean	SD	
**Hyoid bone**	H-Me (mm)	39.53	4.23	39.31	3.87	0.448
	H-PNS (mm)	58.05	6.64	58.43	8.87	0.390
	H-MP (mm)	12.06	5.28	10.99	5.02	0.002^**^
	H-HP (mm)	82.45	7.45	82.39	7.21	0.909
	H-RGN (mm)	32.70	3.86	31.98	3.57	0.014^*^
	H-VP (mm)	15.07	6.68	15.45	6.16	0.259
	H-MSP (mm)	4.10	2.89	3.88	2.67	0.425
	H-C3ia (mm)	31.82	3.95	32.13	4.19	0.155
	H-S (mm)	101.00	8.87	101.16	8.32	0.701
	RGN-S (mm)	104.61	5.92	105.09	5.93	0.003^**^
	RGN-C3ia (mm)	62.98	5.22	62.57	5.47	0.242
	C3ia-S (mm)	97.39	5.67	97.16	5.44	0.269
	Hyoid triangle height (mm)	6.44	3.10	5.79	3.20	0.029^*^
**Craniocervical**	SNL-OPT (°)	82.08	7.34	80.87	7.22	0.005^**^
	SNL-CVT (°)	102.06	6.98	100.92	6.62	0.003^**^
	OPT-CVT (°)	175.14	2.28	175.30	2.51	0.378
	Ba-C3ia (mm)	57.87	3.64	57.99	3.60	0.270
	C0-C1(mm)	16.03	2.58	16.24	2.55	0.299
	C1-C2 (mm)	15.96	2.86	16.13	2.80	0.303

°(degree); SD (standard deviation); mm (millimeters).

^*^P < 0.05; ^**^P < 0.01; ^***^P < 0.001.

The analysis of nasopharyngeal measurements revealed no statistically meaningful differences following treatment. In contrast, oropharyngeal assessment demonstrated a notable reduction in both surface area (from 494.93 ± 110.55 mm² to 480.21 ± 107.83 mm², *P* = 0.021) and sagittal width (from 10.56 ± 2.79 mm to 10.10 ± 2.73 mm, *P* = 0.017) with statistical significance. Regarding hypopharyngeal measurements, further analysis revealed significant decreases in multiple parameters, including surface area (from 185.76 ± 53.87 mm² to 174.98 ± 56.87 mm², *P* = 0.003), volume (from 4957.04 ± 2018.00 mm³ to 4524.50 ± 2318.60 mm³, *P* = 0.006), MCA (from 103.44 ± 57.63 mm² to 86.85 ± 44.58 mm², *P* = 0.001), and sagittal width (from 12.74 ± 3.03 mm to 11.97 ± 3.30 mm, *P* = 0.014). Although total pharyngeal measurements also showed a decrease, the extent of change was insufficient to attain statistical significance.

Regarding the HB measurements, the analysis revealed statistically significant post-treatment reductions in the H-MP distance (from 12.06 ± 5.28 mm to 10.99 ± 5.02 mm, *P* = 0.002), H-RGN distance (from 32.70 ± 3.86 mm to 31.98 ± 3.57 mm, *P* = 0.014), and hyoid triangle height (from 6.44 ± 3.10 mm to 5.79 ± 3.20 mm, *P* = 0.029). In contrast, the RGN-S distance exhibited a significant increase (from 104.61 ± 5.92 mm to 105.09 ± 5.93 mm, *P* = 0.003). For CC posture measurements, the pre- and post-treatment analysis demonstrated a statistically significant reduction in the SNL-OPT (from 82.08 ± 7.34° to 80.87 ± 7.22°, *P* = 0.005) and SNL-CVT angles (from 102.06 ± 6.98° to 100.92 ± 6.62°, *P* = 0.003) post-treatment. Conversely, the C0-C1 and C1-C2 distances showed slight increases, though these changes were not statistically relevant.

## Discussion

TMD is a common disorder of the oral and maxillofacial regions, with multifactorial yet debated causes^20^. Noninvasive treatments include occlusal splints, physiotherapy, manual therapy, psychotherapy, counseling, and medications^21^. Among these, SS are a widely used, effective noninvasive option. While dental appliances influence PA, HB, and CC posture, research on SS is limited. This study primarily evaluates 3D changes in PA and secondarily examines HB and CC posture following SS treatment in adult TMD patients.

Regarding the primary outcome variables, this study did not find any significant changes in the nasopharyngeal space. As the skeletal bony structure supports the nasopharyngeal space^22^it may remain unaffected by changes in mandibular position post-treatment, resulting in no meaningful variation in its volume. In contrast, Ulusoy et al.^23^ investigated how functional correction of Class II with an activator affects PA dimensions over time, demonstrating alterations in the nasopharynx. The oropharyngeal sagittal width significantly decreased post-treatment, likely due to mandibular backward movement and rotation, as seen in our prior study^24^. Zhao et al.^25^ noted jaw position shifts after using the SS, possibly affecting the airway, and reported that the SS, with a thickness of approximately 2 mm, passively increased the inter-jaw distance, induced clockwise mandibular rotation, and raised the mandibular angle. Dadgar-Yeganeh et al.^26^ linked high-angle facial profiles with smaller minimal airway cross-sections and TMD history. Similarly, Derwich et al.^5^ observed reduced oropharyngeal width and downward-backward mandibular rotation with an occlusion splint and physiotherapy.

The present study found statistically significant alterations in the hypopharyngeal airway’s volume, surface area, MCA, and sagittal width after SS therapy. Due to the lack of rigid skeletal support, the oropharyngeal and hypopharyngeal airways are prone to soft tissue collapse, whereas the nasopharynx is more stable because of its surrounding skeletal structures^22^. Occlusal splint treatment can induce a mandibular and condylar clockwise rotation, moving the mandible backward and downward. This may alter pressure within the PA, passively compressing and elongating the pharyngeal wall^27^. For the total pharyngeal surface area, volume, and MCA, a slight but statistically insignificant decrease was observed. Elfeky et al.^28^ found that TWB appliance treatment in growing Class II malocclusion patients significantly increased PA dimensions and altered HB position. Similarly, Bavbek et al.^29^ reported that FFRD improved both PA size and HB positioning, promoting forward displacement.

Although statistically significant reductions in oropharyngeal and hypopharyngeal airway dimensions were observed following SS therapy, the clinical significance of these changes remains unclear. Recent studies have shown that airway narrowing beyond specific volumetric and cross-sectional thresholds is associated with increased risk and severity of OSA^30^. However, the magnitude of airway reduction in this study did not reach levels typically linked to clinically significant obstruction. Nonetheless, even modest airway decreases could impact patients with predisposing factors such as obesity, craniofacial anomalies, or existing OSA^31^. Therefore, comprehensive respiratory and sleep evaluations are recommended before SS therapy, especially in at-risk individuals. Future prospective studies incorporating functional respiratory assessments such as polysomnography are warranted to clarify the clinical impact of these anatomical changes^32^. Clinicians should evaluate respiratory status before SS therapy, as it may reduce pharyngeal dimensions, rendering it unsuitable for OSA patients or those at risk^5^.

With respect to HB positional changes, this study found that SS therapy led to a significant decrease in H-MP, H-RGN distances, and hyoid triangle height, and an increase in RGN-S distance, likely reflecting mandibular downward and backward displacement and clockwise rotation post-treatment, as noted previously^24^. According to the literature, during mandibular alterations, the HB position is likely to maintain stability, which may be attributed to muscle remodeling and compensatory contractions anchoring the HB^33^. Additionally, the HB tends to stabilize to preserve the PA, especially in patients at risk for OSA^34^maintaining a stable relationship with the cervical vertebrae. Although significant changes in hyoid-related measurements were observed in our study, it remains unclear whether these reflect active displacement of the hyoid bone or passive shifts secondary to mandibular repositioning. The absence of reference measurements relative to stable skeletal landmarks limits definitive interpretation. Future studies incorporating such stable anatomical references are necessary to determine whether the hyoid bone undergoes active displacement or passive adaptation during SS therapy. A previous study on 40 TMD patients undergoing long-term splint therapy and physiotherapy found significant HB lowering via cephalometric analysis. These differences between study findings highlighting the complex nature of HB dynamics in TMD treatment and the influence of therapy duration, methods, adjunct treatments, and individual variability^5^.

Regarding CC posture measurements, this study observed significant reductions in the SNL-OPT and SNL-CVT angles, reflecting a transition from forward head posture to improved backward cervical alignment post-treatment. These results align with prior studies showing posture improvement in TMD patients after occlusal splint therapy, assessed via posturo-stabilometric analysis^35^. Cervical vertebra inclination has been linked to PA dimensions, with greater anterior lower angles of C2 and skull base angles associated with wider Pas^36^. Soft tissue tension and neuromuscular feedback influence the relationship between PA, head posture, and craniofacial morphology, affecting CC angulations. While forward head posture may improve PA in some cases, it may hinder it in others^37^. In this study, C0–C1 and C1–C2 distances increased, though without statistical relevance. Kang et al.^19^ reported that TMJ osteoarthritis could alter head posture due to reduced airway space following SS treatment. Proper CC alignment is vital for system integrity, as occlusal instability may disrupt morphostatic balance and muscle tension, potentially alleviating neck pain and enhancing posture in TMD patients^38^. Minervini et al.^39^ suggested TMD may originate from postural dysfunction affecting CC-supporting muscles. Thus, maintaining CC postural balance is essential for optimal function, with muscle activation supporting jaw alignment and physiological centric relation.

The present study findings do not diminish the importance of SS therapy; rather, they highlight the need for pre-treatment respiratory assessments, especially for individuals who may be more susceptible. Additionally, these results help us to make more accurate diagnoses, adopt appropriate treatment plans, and ensure stable and precise outcomes by considering the intricate relationship between mandibular position, airway patency, and postural adaptation in TMD management. One limitation of the current study is the absence of a control group due to its retrospective design and ethical concerns regarding TMD patients, which limits the ability to draw direct comparisons between the treatment and non-treatment conditions. Additionally, a follow-up long-term study for assessing the long-term effects of the intervention is recommended to better understand the sustainability and enduring impacts over time. Furthermore, although the FHP was used to standardize head positioning during CBCT acquisition, it may not fully reflect physiologic posture. The natural head position (NHP) is considered more representative in airway and CC studies; thus, future research using NHP may provide additional clinically relevant insights.

## Conclusion

This study revealed that SS therapy in adult TMD patients significantly reduced oropharyngeal and hypopharyngeal airway dimensions, which may be due to mandibular backward displacement and clockwise rotation. However, the nasopharyngeal dimensions remained unchanged. The HB exhibited positional changes, and CC posture improved with reduced forward head inclination. Although these airway changes were statistically significant, their clinical relevance remains unclear. They may still be relevant in individuals with predisposing factors such as existing OSA risk. While SS therapy may have an impact on the respiratory system or certain other factors, it is still considered a common, beneficial, non-invasive treatment for TMD, as demonstrated in prior research and our previous studies.

## Supplementary Information

Below is the link to the electronic supplementary material.

Supplementary Material 1

## Data Availability

The data that support the findings of this study are available from the corresponding author upon reasonable request.

## Abbreviations

SS: Stabilization splint
TMJ: Temporomandibular joint
TMD: Temporomandibular disorders
CBCT: Cone-beam computed tomography
3D: Three-dimensional
PA: Pharyngeal airway
HB: Hyoid bone
CC: Craniocervical
DC/TMD: Diagnostic criteria for temporomandibular disorders
