# Supplementary material for: Three-dimensional assessment of pharyngeal airway hyoid bone and craniocervical changes after stabilization splint treatment in temporomandibular disorder patients
Source: Sci Rep. 2025 Sep 25;15:32874. doi: 10.1038/s41598-025-17583-0 (PMC12464249; doi:10.1038/s41598-025-17583-0)
Supplement: Supplementary file 1 — Supplementary Material 1 [file 41598_2025_17583_MOESM1_ESM.docx]

**Supplementary Figures with Captions**


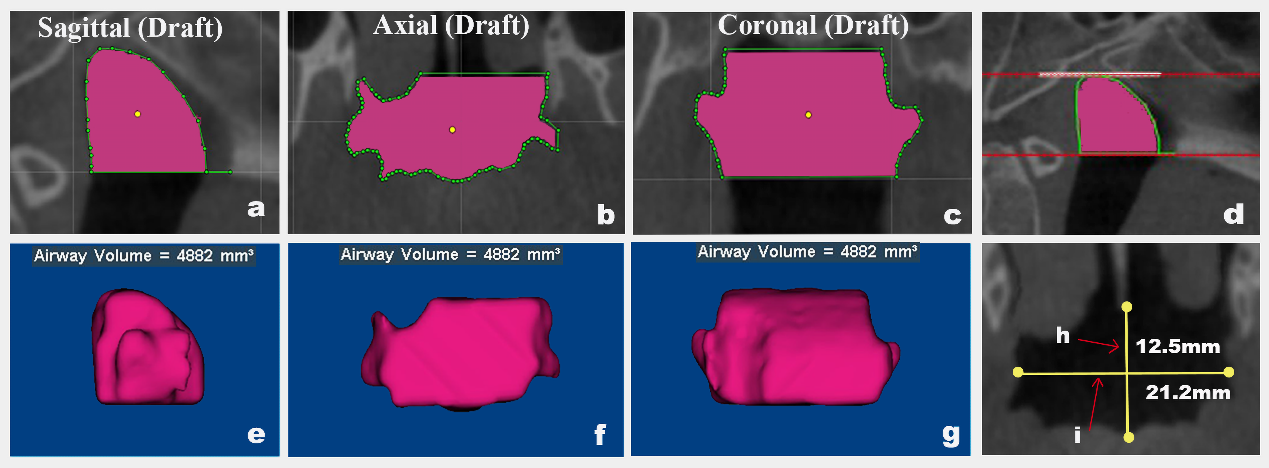


**Supplementary fig.1** **Nasopharyngeal airway:** (a) Sagittal view of the surface area; (b) Axial view of the surface area; (c) Coronal view of the surface area; (d) Multiplanar view of the airway area and MCA; (e) Sagittal view of the airway volume; (f) Axial view of the airway volume; (g) Coronal view of the airway volume. (h) Sagittal width of nasopharyngeal NP (A/ P); (i) Lateral width of nasopharyngeal NP (R/L). (images generated using Dolphin 11.95; https://www.dolphinimaging.com)


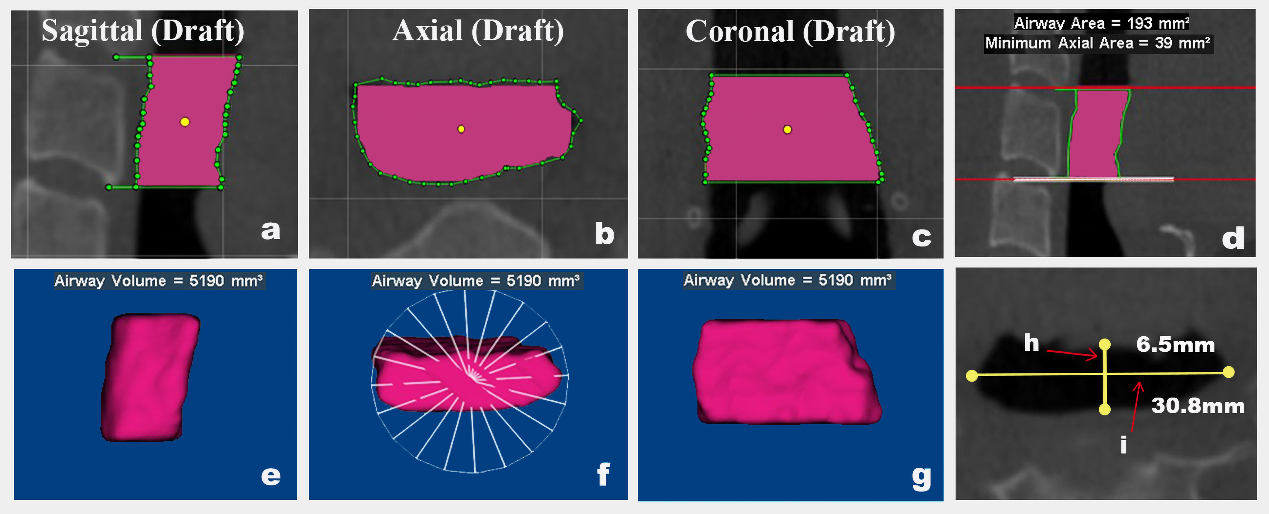


**Supplementary fig.2 Hypopharyngeal airway:** (a) Sagittal view of the surface area; (b) Axial view of the surface area; (c) Coronal view of the surface area; (d) Multiplanar view of the airway area and MCA; (e) Sagittal view of the airway volume; (f) Axial view of the airway volume; (g) Coronal view of the airway volume. (h) Sagittal width of hypopharyngeal HP (A/ P); (i) Lateral width of hypopharyngeal HP (R/L). (images generated using Dolphin 11.95; https://www.dolphinimaging.com)


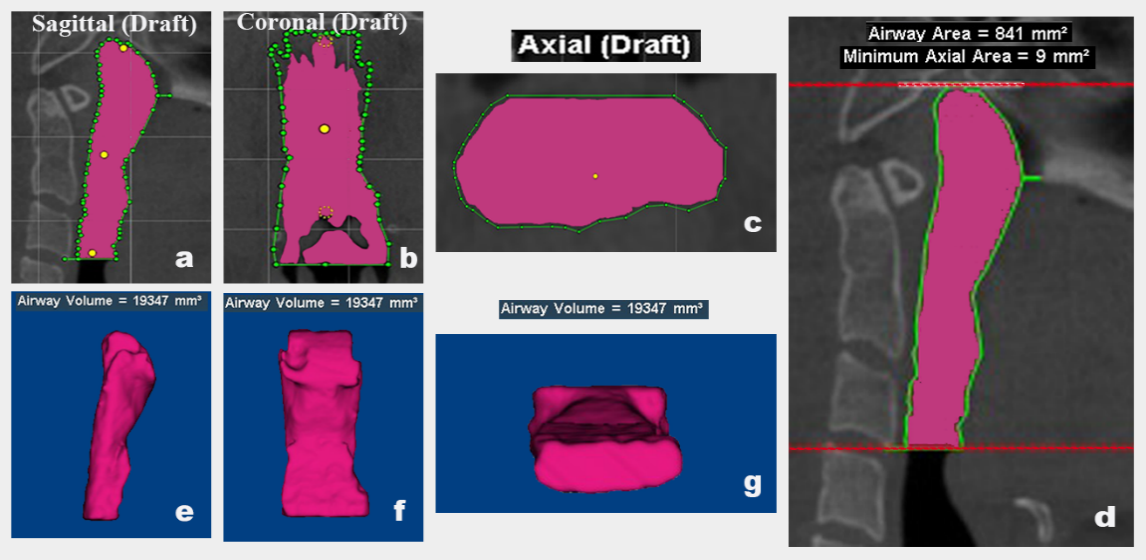


**Supplementary fig.3 Total pharyngeal airway:** (a) Sagittal view of the surface area; (b) Axial view of the surface area; (c) Coronal view of the surface area; (d) Multiplanar view of the airway area and MCA; (e) Sagittal view of the airway volume; (f) Axial view of the airway volume; (g) Coronal view of the airway volume. (images generated using Dolphin 11.95; https://www.dolphinimaging.com)
